# Sphenoid sinus hyperpneumatization: anatomical variants, molecular blueprints, and AI-augmented roadmaps for skull base surgery

**DOI:** 10.3389/fendo.2025.1634206

**Published:** 2025-09-18

**Authors:** Andra Ioana Baloiu, Florin Filipoiu, Corneliu Toader, Razvan-Adrian Covache-Busuioc, Octavian Munteanu, Matei Serban

**Affiliations:** 1 Doctoral School, “Carol Davila” University of Medicine and Pharmacy, Bucharest, Romania; 2 Department of Anatomy, “Carol Davila” University of Medicine and Pharmacy, Bucharest, Romania; 3 Department of Neurosurgery, “Carol Davila” University of Medicine and Pharmacy, Bucharest, Romania; 4 Department of Vascular Neurosurgery, National Institute of Neurology and Neurovascular Diseases, Bucharest, Romania; 5 Department of Medical Research, Puls Med Association, Bucharest, Romania

**Keywords:** hyperpneumatization, skull base anatomy, transsphenoidal surgery, cerebrospinal fluid leakage, neurovascular injury, advanced imaging, artificial intelligence

## Abstract

The sphenoid sinus is a complex part of the skull base that has a high degree of anatomical variation, the most interesting of which occurs with hyperpneumatization, in which pneumatized air cells extend beyond their normal limits into the clivus, pterygoid processes, and sphenoidal wings. These hard to note hyperpneumatized imaging variants are disregarded in routine imaging but have potential to grossly alter important neurovascular landmarks, which is a challenge for the precision and safety of transsphenoidal surgical approaches. In this review, we provide an exten- sive, state-of-the-art investigation of sphenoid sinus hyperpneumatization, synthesizing novel pri- mary research discoveries with primordial radiological, anatomical, and clinical intrepidity. Our exploration to unravel the embryological basis for sinus development elicits an intricate balancing act between osteoclastic activity and the myriads of molecular actors such as RANKL/OPG, SHH, and BMP signaling pathways that delineate pneumatization in the skull base system. We demon- strate via in-depth radiological analysis how high-resolution CT (HRCT), dual-energy CT (DECT), and 7T MRI furnish unparalleled visualization of these variants, allowing identification of involved thinned bony walls, dehiscent canals, and high-risk zones for neurovascular insults. Clinically hy- perpneumatization is not just an anatomical curiosity, it may foreshadow operative complications and neurological symptoms. We discuss how it complicates endoscopic transsphenoidal ap- proaches and may increase the risk of internal carotid artery (ICA) injury, optic nerve impingement, and cerebrospinal fluid (CSF) leak. Surgical advances such as AR/VR-assisted neuronavigation and hydroxyapatite-based skull base reinforcement techniques are explored for their potential to de-risk these procedures and improve outcomes. Proactively, we propose that the future of sphenoid sinus hyperpneumatization research be one that adopts AI-driven morphometric analyses, clinically standardized classification systems, and longitudinal clinical studies to dissect its pathophysiolog- ical mysteries. This paper aims to develop an understanding of this omitted but clinically important anatomical variant by integrating basic anatomical principles with technology in order to provide clinicians, researchers, and surgical teams with a more nuanced, applicable exploration of the topic.

## 1 Introduction

### 1.1 Background and rationale

The sphenoid sinus is an air-filled space encased within the sphenoid bone in the skull base, located at the sella turcica and pituitary gland inferiorly, anteriorly to the clivus, and laterally to the carotid sinuses (1). Due to its close anatomical relationship to critical neurovascular structures including the internal carotid arteries (ICAs), optic chiasm, and cranial nerves that run through the cavernous sinus, the sphenoid sinus is of extreme importance in the transsphenoidal neurosurgical approach but can inherently pose risk in some individuals due to anatomical variants with large amounts of pneumatization (2).

Sinuses begin forming during the fourth month of gestation when epithelium invades the presphenoid cartilage. This process is ongoing into childhood, and into early adulthood, extending posterolaterally and inferiorly (3). Most sinus areas extend to the sella turcica in adulthood, while some develop into adjacent structures such as the clivus, pterygoid processes, or greater wings, forming large irregular cavities with an area of air space. In these scenarios, the sinus may be associated with thinned walls, or even completely atrophied and possessing no bony wall, increasing the risk to surrounding structures with instrumentation (4). Moreover, septal asymmetries are frequent, and in some circumstances, the intersinus septa can deviate off the midline and insert onto the optic or carotid canals, creating another level of operative complexity, notwithstanding the majority being asymptomatic (5).

Pneumatized patterns of the sphenoid sinus can be described as conchal, presellar, sellar or postsellar dependent on the relationship of the sinus to the sella turcica. While conchal variants feature compact bony coverage, sellar and postsellar variants progressively expose neighboring structures as wall attenuates. Overdeveloped sinuses often demonstrate complicated and confusing septal anatomy with variable septal presence resulting in decreased confidence for orientation during the endoscopic approach—and increased risk of iatrogenic injury (6).

On imaging, these spaces may resemble pathological processes like tumor erosion, ostelytic lesions, or chronic sinusitis with associated bone remodeling. Although high-resolution computed tomography (CT) is the primary imaging modality for visualizing cortical anatomy, septal insertions, and dehiscence; magnetic resonance imaging (MRI) provides pertinent information about adjacent soft tissues, especially the optic apparatus and pituitary complex (7, 8). Discerning physiological hyperexpansion from pathological remodeling requires careful review of cortical thickness, bony margins, and adjacent soft tissue findings.

From a neurosurgical perspective, hyperexpanded sinuses may provide increased surgical access; however, they leave the anatomy more unpredictable. Thinner bony walls, displaced septa, and changes to normal surgical landmarks may diminish orientation or directly expose sensitive structures (9). Therefore, endoscopic neuronavigation and intraoperative imaging have become important adjuncts to help maintain surgical orientation and minimize complications (10). In the clinical setting, hyper-expansive sinuses have also been linked to retro-orbital discomfort, barotrauma, and spontaneous cerebrospinal fluid (CSF) leaks—albeit a precise pathophysiological explanation is still being elucidated (11).

This review not only aims to integrate the anatomical, radiological, and operative viewpoint but also offers a new conceptual approach to understanding sphenoid sinus hyper-expansion—not just in geometrical extent but fragility, neurovascular exposure, and intraoperative uncertainty. Although based on existing morphologic classification, our approach intends to emphasize clinically relevant actionable risk types (1). The rationale for this work is detailed in the final section of the review where we propose a functional-surgical model to advance the preoperative individual assessment and procedure planning approach.

### 1.2 Aim of the review

This review aims to present a evidence-based synthesis of the existing knowledge of sphenoid sinus hyperpneumatization with a particular focus on its developmental basis, anatomical variability, radiological appearances, and neurosurgical relevance. We hope to provide clinical information from imaging studies, anatomical studies, and operative reports that define the clinical characteristics of this frequently under-appreciated CT variation.

To do so, we first evaluate the embryological and post-natal processes of sphenoid sinus pneumatization, illustrate the various morphologic subtypes representing excessive expansion, and evaluate the implications of the morphologies for both standard and complicated transsphenoidal neurosurgical approaches. We examined various classification schemes that have defined hyperpneumatized sinuses, examining their clinical utility, as well as their limitations.

We highlight the radiological features of particular concern that can complicate diagnosis or create surgical dilemmas, including septal anomolies, dehiscent walls and important neurovascular relationships, provide a summary of noted complications available in the literature, and identify possible anatomical predictors of unfavourable surgical outcomes.

Finally, we present future relevant areas of research - such as creating consensus radiologic criteria, studying the biomechanical properties of expanded sinus bone, and augmenting virtual reality applications for surgical planning. We intend to provide a translational construct of radiologic and surgical planning and anatomical science to provide a clinically relevant perspective to both clinicians and researchers.

## 2 Anatomical and embryological overview

### 2.1 Development of the sphenoid sinus

The sphenoid sinus is a paranasal air cell and is located in the body of the sphenoid bone, at the base of the skull in a midline position and is clinically and structurally important because of its location to significant neurovascular structures (12). The sphenoid sinus develops through changes in the sphenoid bone, as an embryologic outpouching becoming a multicompartmentalized chamber in the presence of pneumatisation and septation; sought of an outcome interplay of genetic predispositions, molecular signaling pathways, and environmental influences (13). Generally, the timing of development is fairly consistent, however hyperpneumatized variants are clinically important due to differences in size, bone quality, and morphology, all of which create challenges in respect to differential diagnostics and surgical risk (14). Embryologically, the sphenoid sinus develops from posterior ethmoidal air cells that evaginate out of the sphenoethmoidal recess by way of nasal epithelial invaginations causing a mesenchymal stem cell transition to osteoclast-like activity to resorb cartilage at the presphenoid, collectively from epithelial-mesenchymal signaling pathways (15). For example, the sonic hedgehog (SHH) cascade up-regulates epithelial proliferation due to PTCH1-SMO activation, while bone morphogenic proteins (BMPs) stimulate SMAD-governed bone resorption and bio-organized directional growth of the sinus cavity (16).

The Wnt/β-catenin signaling can also stimulate osteoblast differentiation and relevant ossification of bone, by way of LRP5/LRP6 activation (17). A reduced Wnt/β-catenin signaling cascade is associated with mutations in RNA binding motif (Rbm) proteins (RUNX2) and abnormal cranial bone development. Likewise, fibroblast growth factors (FGF) 2 and 10 provide incidental support to continue proliferation of the mucosa, and developing blood vessels importantly needed for sinus cavity expansion (18). Also, mutations in fibroblast growth factor receptors (FGFR1/2) are involved with sphenoid morphology changes through syndromic craniosynostoses (19). Histologically, the sinus walls are transformed from immature woven bone to mature lamellar bone by endochondral ossification; and at various times screens even from hyperpneumatization variants, demonstrate the increased RANKL expression in mucosa to increase osteoclastogenesis leading to softened bone, exquisitely thin over the overlying ICAs and optic nerves, increasing the risk for intracranial bleeding during surgery (20). Postnatally, the expansion occurs in two phases. Phase one is approximately 2-4 years of age, presumably suggests an increased nasal airflow and mucosal proliferation. Studies using computational fluid dynamics (CFD) have reported the high-velocity turbulent flow in the sphenoethmoidal recess creates levels of shear stress in the cavity that as a result of the activation of Piezo1 ion channels in osteocytes to recruit osteoclasts for bone-resorbing (21, 22) Phase two corresponds with puberty and adolescence, when sex hormones, GH and IGF-1, ignite synergistic further expansion posterior to the clivus, leading to probable poor quality bone metabolism and bone turnover (23).

In summary, the overall development leads to four patterns of pneumatization, conchal, presellar, sellar and postsellar (24). Conchal would be believed to be extremely rare with notable thickness of bone and would frequently occur with craniofacial dysmorphism. Sellar and postsellar variations have been reported to occasionally distally expand posteriorly with postsellar typically reported to extend posteriorly to the clivus, dorsum sellae or pterygoid processes. There have been reports of sphenoid sinus extending to anterior margin of cranial base (foramen magnum) demonstrate raises questions on what that means for stability & structural integrity of the cranial base (13).

Biomechanically, it has been postulated that *in vivo* pneumatised cavities could modulate stresses during mastication or mechanism trauma. With the idiosyncratic dissemination of stresses, such a variation would predispose the cranial base to: stress corrosion, microfractures, spontaneous CSF leaks into the middle ear cavity, and spontaneous pneumocephalus (25). With those considerations a question could be posed from a developmental evolutionary tint because comparative literature demonstrated non-human primate species with smaller floatable and non-pneumatized variations of the sphenoid sinus cavity. In contrast, comparative evidence proposes humans advantageously with greater spatial expansion (pneumatization) of the sphenoid sinuses promotes increased respiration efficiency; and a posteriorly rotated base of the skull to suggest our encephalization process is indeed inverting (26, 27).

### 2.2 Relevant neurovascular relationships

Sphenoid sinus disease is clinically complex due in part to an anatomical relationship with important neurovascular structures. The sphenoid sinus posterior wall is in direct contact with the clivus and dorsum sellae; the superior wall, vagary the sphenoid sinus, forms the floor of the sella. A transsphenoidal corridor exposes the pituitary gland with cavernous sinuses on either side containing the ICAs, optic nerves, cranial nerves III, IV, VI, and V1 and V2. Extreme hyperpneumatization can change the size and configuration of these compartments leading to pre-operative variations that can have implications upon any surgical approach to the sphenoid (28).

The most concerning elements are the ICAs and particularly so if the normally present thin bony lamella separating the aircraft noses from the sphenoid sinus has flattened or been absorbed altogether. Bony dehiscence of the ICA canal has been demonstrated to exist in almost 25% of hyperpneumatized cases on high-resolution CT angiography greatly increasing the opportunity for vascular injury when attempting to access the sella turcica or pituitary fossa utilizing a transsphenoidal approach (29). Additionally, in some patients, there may be a fenestration of the ICA, as a normal structure when the vessel divides and rejoins intraluminally, that may coexist with hyperpneumatized coverage and thereby obstruct the surgical pathway and could significantly obscure landmark structures with irregularities in the wall and traditional endoscopic visualization. Similarly, optic nerves are also at risk. There is rupture at the optic canal in 15-20% of hyperpneumatized instances of sphenoids, and when this occurs the nerve is positioned directly within the air cavity. The likelihood of trauma to the surgical instruments as well as additional risk for compressive trauma from inflammatory changes within the sinus. These normal anatomical references such as the opticocarotid recess (OCR)—the place where the optic and carotid canals meet—may be distorted in these variants as previously defined by severity making surgical navigation less intuitive (30).

The cavernous sinus located laterally to the sphenoid consists at the least a complex venous plexus along with multiple cranial nerves. It is plausible that hyperpneumatization may create an overabundance of air cells in the lateral wall that may push or restrict neural components. Rarely, the abducens nerve may protrude into the sinus cavity which not only provides a surgical quandary but may also be a finding recognized as incidental (31). The internal constituents of the sinus also rely on many of the overall endoscopic pictures, still somewhat difficult to depict successfully in the last series of hyperpneumatized examples. Typically, the intersinus septa arise from the midline; however, in the hyperpneumatized examples from the presentations, they are often asymmetrical either to the ventral surface of the ICA or optic nerve. The concern in the course of an endoscopic procedure is that the operating surgeon may violate and disrupt a midline septa: leading to a range of bad outcomes, possibly even lacerating the ICA. There are examples of septal avulsion injuries in the literature that reiterate the necessity to have some awareness of the anatomy being encountered (32). Recent technology has captured new imaging technologies to not only become aware and identify variant sinus morphology, but to also maintain operational awareness. Radiomic analysis may allow the surgeon to quantify a limited number of textural and geometric features from CT datasets and thereby provide further information on the morphology or integrity of bony walls. Artificial intelligence (AI) algorithms trained on multiple craniofacial imaging datasets can now also algorithmically predict hyperpneumatizations based on skull base and craniofacial geometries. When these technologies are coupled with augmented rationality (AR) platforms, the opportunity to create real-time patient specific anatomical overlays provides a superior level of surgical action and consequentially facilitates a tailored patient pre-operative risk assessment (33).

In conclusion, the sphenoid sinus acts like a crossroads among developmental biology, craniofacial biomechanics, and evolutionary adaptation. Hyperpneumatized anatomical variants often remain silent; however, they naturally present variability that comes to an end in clinical/operative challenges and difficulties intraoperatively. Appreciation of developmental/vertricular sequence, development morphology incorporated to significant relationships with neurovascular structures is necessary information for limiting potential iatrogenic harm and obtaining successful treatment outcomes for this complex anatomical area (34).

## 3 Definition and classification of hyperpneumatization

### 3.1 Criteria for Hyperpneumatization

Hyperpneumatization of the sphenoid sinus is defined as an excessive, anatomically significant protrusion of air-filled cavities beyond physiological limits, frequently passing through adjunct structures like the clivus, pterygoid (PP), dorsum sellae, and in extreme presentations even the basioccipital (35).Although it stilloccurs normally via usual pathways of pneumatization, this excessive development can rupture neurovascular barriers, thin or eat away various courses of bony segments and result in a different direction of approach to access these areas during surgery on the skull base or management of the pathologically affected sphenoid sinus (36). Current assessment is predominantly through radiological relationships to known anatomical markers. Usually, pneumatization of the sphenoid sinus is limited anteriorly of the clivus with posterior limits being at the level of the dorsum sellae. In hyperpneumatized variants however, there are air cavities marginally beyond the dorsum sellae or occasionally extending backwards to the level of the anterior occipital bone foramen magnum (37). A common criterion cited in CT studies is one where air cells extend into areas > 10 mm posterior to the posterior edge of the sella turcica. There is frequently a concomitant volumetric expansion of greater than 15 cm³ in adults following population morphometric analyses broken down by age, sex, and existence of craniofacial structure (38, 39). Hyperpneumatization can also be identified by involvement of adjacent bony markers - e.g. posterior extension into the clivus, lateral pneumatization of the pterygoid processes and sphenoid body thinning. Bone densitometry in these above areas frequently shows cortical bone thinning of < 0.5 mm based on high definition CT images (40). In severe instances, there may be no lamellae that protect the optic nerve canals or ICA, resulting in direct exposure of these structures. This exposure increases risk for surgical complication through neurovascular injury and CSF leaks (41).

MRI contributes complementary evidence to the clinical picture for the assessment of anatomical considerations of borderline bony dehiscences or soft tissue changes. An example of this is assessment of mucosal thickening with T2 sequences, seeing this with inflammatory or barobasic modifications within hyperpneumatized sinuses (42). Diffusion-weighted imaging (DWI) and magnetic resonance angiography (MRA) can provide additional information about the adjacent neurovascular structures, that can be interpreted with CT data (43). Ultra-high-field 7T MRI adds further value by assessing the microtrabecular architecture of the bone and could distinguish between non-cynical (benign) hyperpneumatization, versus osteolytic or neoplastic processes (44). Pathophysiologically, hyperpneumatization is postulated to occur from deregulated balance between osteoblastic-osteoclastic factors. Enhanced airflow through the nares and local changes in mucosal inflammation, along with a background of abnormal craniofacial biomechanics are believed to denote osteoclastic activity during increased sinus expansion (45). CFD studies of turbulent flow and anatomy of the sphenoethmoidal recess demonstrate that the shear stress of air turbulence in this area activates the Piezo1 channels in osteocytes potentially stimulating bone remodelling (46). Inflammatory mediators cytokines e.g. IL-6 and TNF-a have been implicated in modulation of the mucosal environment and reconstruction. Polymorphisms in RUNX2 and FGFR2 genes are implicated in predisposing excessive development of the sinus, especially in patients with craniofacial syndromes or abnormal bone metabolism status (47).

### 3.2 Patterns of pneumatization

Traditionally, the pneumatization of the sphenoid sinus is categorized based on relative location of the sinus cavity and the sella turcica: conchal, presellar, sellar, and postsellar. However, imaging studies have revealed a larger morphological cohort that includes accessory air cells and unusual expansions that bypass classical classification. Recognizing these anatomical variations is important, especially in the neurosurgical setting where sinus architecture is involved in designing preoperative approaches and intraoperative hazards (48, 49). The conchal type is the most perplexing. It consists of a small, shallow air space anterior to the sella turcica, limited to the presphenoid. It is often observed in those with craniofacial deformities such as craniosynostosis or mid-face hypoplasia which are associated with faulty SHH signaling during development. Owing to the thick bony walls and lack of internal sinus landmarks the conchal pattern is the most challenging surgical access, even with the aid of CT (50–52). The presellar type has air cells extending to the anterior border of the sella but not invading the sellar floor and represents a transitional stage of pneumatization that is typically seen in the younger population or individuals with delayed skull base maturation. This also has the potential to distort the anticipated anatomical configurations based on the unpredictability of variable presence or absence of septal insertions. Based on population studies it has a reported frequency of between 20-30%, with an average volume of 6-9 cm³ (53). The sellar type has air cells expanding beneath the pituitary fossa, under the sella turcica. This variant occurs in roughly 60-70% of adults and has the same benefits for surgical access, but typically results in some thinning (probably also dehiscence) of the sellar floor with a subsequent risk of CSF leak, as well as change in landmarks close by, including the OCR (35).

The postsellar pattern, accompanied by hyperpneumatization, can extend into the clivus, dorsum sellae, or pterygoid processed (the pterygoid recess). The postsellar pattern is present in 15-20% of adults, accompanied by thinning at the critical wall—clival recess, wall just lateral to the cavernous sinus, etc. Septal insertions onto ICA canals are particularly problematic during transsphenoidal resection (54). In addition to the canonical types, many additional accessory concepts of pneumatization variability have been identified using high-resolution imaging. The pterygoid recess will displace the vidian nerve and occupy space in the pterygopalatine fossa during a postsellar pattern of pneumaticity. The clival recess extension can include the anterior occipital bone; and rare reports of lesser-wing pneumatization associated with retro-orbital symptoms suggest the possibility of obstructed access to the anterior skull base (55, 56). Biomechanical modeling, especially finite element analysis (FEA), models suggest that extensive pneumatization has an impact model on cranial base biomechanics and loading (pneumatization redistributes cranial base stress). These sinuses may act as stress amplifiers upon impact loading, and in the context of cranial base stress, may be involved in spontaneous CSF leaks or even microfractures at sites of cortical thinning. The clinical findings presented subsequently are based on the supposition that there is a biomechanical component described here; most support the notion that hyperpneumatized variants are associated with significant complication rates (57, 58).

An evolutionary perspective may offer some insight into the variant morphology of sphenoid sinus expansion with cranial base flexion and different model of encephalizating Homo sapiens. Whereas non-human primates (gorillas, chimpanzees) have a very low-end ethmoid-sinus system, the anatomy of the sphenoid complex indicates that humans have a more complex, larger, and variable set of sphenoidal complexes (and cavities). The anatomical shifts in these soft-tissue structures were likely functional adaptations permitting greater oxygenation and price approximation of the brain in a bipedal species (59, 60). From a clinical standpoint, being able to tell between patterns will be important for risk stratification, when planning the procedure: pneumatisiational variants of postsellar or accessory type will require further imaging expectations and even a plan to proceed navigating intraoperatively. As imaging technology has improved, the number of studies showing efficacy coupling 3D CT reconstruction with AR -assisted navigation - or relying on radiomics algorithms - have greatly aided in pre-operatively classifying configurations at risk. Future studies should focus on genetics and molecular behavior underlying genetic and singular corporativisms of incidental sinus. Understanding the relationship between genetic make-up of the biophysicical bio-chemical, structural properties of variance will lead to individualized surgical methods based on risk stratification of critical configurations, and possibly, to early identification as a high-risk patient group (61, 62).

## 4 Radiological assessment

### 4.1 Diagnostic imaging modalities

Investigation of sphenoid sinus hyperpneumatization is most useful with a multi- modal imaging assessment that can accurately distinguish normal from abnormal ana- tomical variations. The need for advanced imaging techniques to improve diagnostic accu- racy and surgical planning stems from the proximity of the sphenoid sinus with neuro- vascular structures (e.g., optic nerves, ICAs and cavernous sinuses) and variability in structures due to hyperpneumatization (2, 63). Advancements in technologies in CT and MRI modality in the last few decades have succeeded in visualizing such anatomical var- iations precisely and reliably (64).

For evaluation of normal sphenoid sinus anatomy, high-resolution CT continues to be the gold standard owing to its unique ability to image fine bony structures at submil- limeter resolution. State-of-the-art multidetector CT (MDCT) machines, particularly 64- slice or higher types, provide isotropic voxel acquisition with slice thicknesses of as low as 0.5 mm and offer well-defined cross-sectional images of the sinus cavity (65). Imaging protocols are generally performed at 120 to 140 kVp tube voltages and tube currents of about 150–250 mAs, for optimized cranial base imaging that balances tissue contrast vs radiation exposure (66). The overlapped datasets are reshaped by MEEs with state-of-the- art algorithms such as adaptive statistical iterative reconstruction (ASIR) and model-based iterative reconstruction (MBIR), to preserve details while reducing noise and CT radiation artifacts (67, 68).

The recent focus on dual-energy CT (DECT) in skull base im-aging is likely due to its capability to render bone, air and soft tissues distinguishable based on differences in X- ray attenuation at two energy levels. 80 kVp and 140 kVp DECT protocols enable a better evaluation of hyperpneumatized sphenoid walls, especially for the differentiation be- tween physiological thin cortical bone (which accompanies the pneumatization of the sphenoid) versus pathological osteolysis (69). This is a vital distinction as hyperpneuma- tization could mimic pathologic processes such as chordoma related bone destruction or invasive pituitary adenomas invading into the sphenoid sinus. Radiodensity measure- ments in hyperpneumatized areas typically range from 250 to 400 Hounsfield units (HU) and are markedly different from the osteolytic lesions, which show values below 100 HU due to trabecular bone loss (70–72).

Cone-beam CT (CBCT) has become a valuable adjunctive tool, most notably in an intraoperative setting. CBCT systems were developed for endoscopic skull base surgery to image in real-time at high resolution and with less radiation exposure than conven- tional CT (73). These systems have served a special role in terms of guiding transsphe- noidal procedures, through hyperpneumatized sphenoid sinuses with altered anatomy mitigating standard neuronavigation systems (74). Intraoperative CBCT fused with AR overlays allows for real-time recognition of sinus septations, neurovascular relations, and sites of potential bony dehiscence (75).

Although not effective in characterizing bony anatomy, MRI also is critical in as- sessing the soft tissue structures near hyperpneumatized sphenoid sinuses. High-contrast resolution to evaluate the pituitary gland, cavernous sinuses, and optic nerves is obtained with standard T1- and T2-weighted sequences (76).

In recent years, several innovative methods of constructive interference in steady- state (CISS) or fast imaging employing steady-state acquisition (FIESTA) images have demonstrated advantages in visualization of thin mucosal membranes and the complex path of cranial nerves in the sinus walls (77). DWI has been particularly helpful in detect- ing subtle soft-tissue lesions that may look like hyperpneumatized air cells. Signal heter- ogeneity in mucosal tissues affected by chronic barotrauma, a condition possibly result- ing from the altered aeration patterns in hyperpneumatized sinuses, has been demon- strated in studies evaluating diffusion kurtosis imaging (DKI) (78).

**Figure 1:**
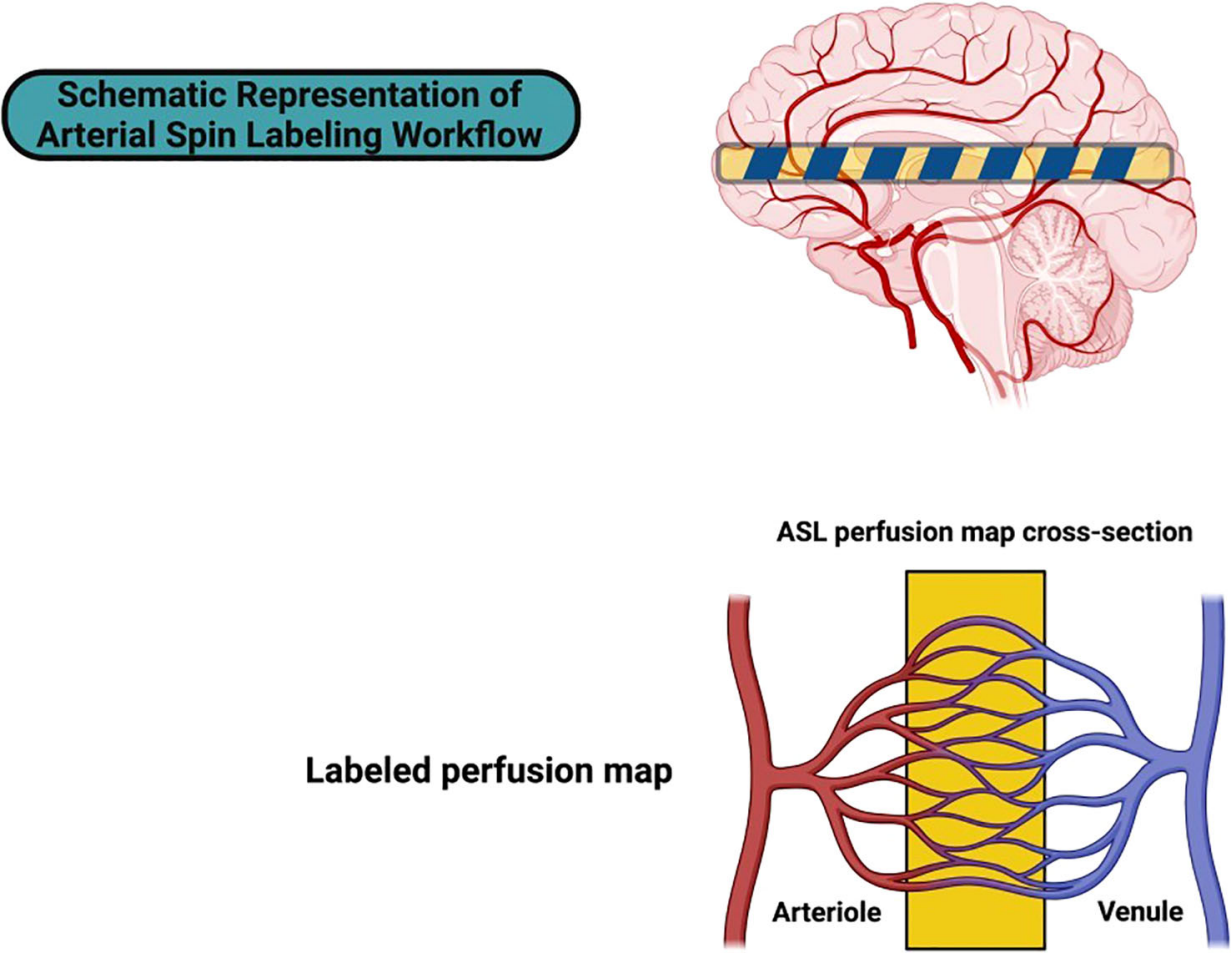
Schematic representation of arterial spin labeling workflow. This figure illustrates the core principles of the ASL technique, a non-invasive MRI-based method for quantifying cerebral blood flow (CBF). The upper portion depicts a sagittal brain view, highlighting the labeling plane (striped region) where arterial blood water is magnetically tagged. This tagging process occurs prox- imal to the cerebral circulation, ensuring that the labeled blood serves as an endogenous tracer for perfusion measurement. The lower section presents a cross-sectional schematic of the ASL perfusion map, emphasizing the transition of tagged arterial blood (red) through the microvascular network into venous circulation (blue). The yellow-highlighted region indicates the imaging plane where signal differences between the label and control images are measured. The resulting perfusion map reflects regional CBF, providing critical insights into vascular function without the need for exoge- nous contrast agents. ASL’s capacity to capture dynamic perfusion patterns has proven particularly valuable in neuroimaging applications, including the evaluation of cerebrovascular reactivity, is- chemic lesions, and neurodegenerative disorders.

Additional functional MRI features, including dynamic contrast-enhanced MRI (DCE-MRI) and arterial spin labeling (ASL) (**Figure 1**), have specifically added new infor- mation related to the vascular dynamics that are expected with hyperpneumatized sinus walls. In fact, data from DCE-MRI studies showed that most patients with hypervascular mucosal patterns, which are usually identified with chronic inflammation, observed less frequently in cases of hyperpneumatized variants (79). This result suggests that the in- creased pneumatization is influenced by balanced osteoclastic activity rather than being a chronic inflammatory process. By providing a measure of tissue perfusion in the absence of exogenous contrast agents, ASL appears to be a valuable method for studying sinus wall vascularization patterns, of particular interest in the evaluation of cases suspected of vascular involvement (80, 81).

### 4.2 Key radiological features

The diagnosis of hyperpneumatized sphenoid sinuses necessitates the awareness of certain radiological features that separates these variants from normal anatomical and pathological processes. The extension of air cells outside normal anatomic boundaries is one of the most diagnostically important features. In normal adult anatomy, the sphenoid sinus has posteriorly extended to the sella turcica but is uncommon to invade the clivus or pterygoid processes (82). However, posterior extension into the clival region on CT im- aging, often with pneumatization reaching the anterior margin of the foramen magnum, is seen in hyperpneumatized variants. Such pneumatization has the appearance of com- plex air-cell constructs with different cortex remodeling (4, 83).

Hyperpneumatized sphenoid sinuses also have another characteristic appearance: bony thinning or dehiscence. Cortical thinning exceeding 50% compared to non-hyperp- neumatized siblings is frequently observed on high resolution CT scans. The thinning of this structure is especially prone to be seen at the sellar floor, OCR, and lateral sinus walls and is secondary to its anatomical configuration and their proximity to the airflow motion within the sinus cavity (84). For hyperpneumatized sinuses, measurements of cortical thickness in these regions commonly are <0.4 mm and in some cases, the optic nerve ca- nals are directly exposed to the sinus cavity, as in complete dehiscence of the region, or the ICAs lie directly adjacent to the sinus cavity (85).

Formation of accessory air-cells is one of the unique imaging features of hyperpneu- matization. Standard pneumatization would generally lead to symmetrical allocation of air-cells around midline, while hyperpneumatized variants may show asymmetric air- cell development (86). The pterygoid recess, for instance, can be pneumatized, pushing air cells laterally into the pterygoid processes, often displacing the vidian nerve, or com- pressing contiguous vascular structures. Clival recess pneumatization is characterized by posterior air cell extension onto the clivus, often associated with cortical thinning adjacent to the basioccipital synchondrosis. Less frequent is lesser-wing pneumatization, which has been correlated with retro-orbital pain due to pressure on the orbital apex (87).

The differentiation of hyperpneumatization from pathological bone erosion contin- ues to pose a significant diagnostic conundrum. Neoplasm or chronic inflammatory con- dition-associated osteolytic lesions can also cause similar cortical thinning when seen along hyperpneumatized sinuses. Hyperpneumatized bone has a smooth, corticated margin, in contract, erosive bone has irregular, shaggy edges with overlying soft tissue infiltration. To differentiate trabecular bone from osteolytic tis-sue according to the en- ergy-dependent attenuation patterns, dual-energy CT has been especially valuable in these situations (88, 89). Both neural and vascular involvement are a radiologically con- siderable risk in hyperpneumatized sinuses. In cases where air cells extend laterally into the sinus wall, cranial nerves III, IV, V1, V2, and VI may be partially or completely ex- posed to the sinus cavity (28). Diffusion tensor imaging (DTI)-based MRI tractography has shown aberrant trajectories of the optic nerve and maxillary nerve pathways in hyperp- neumatized variants. Particularly, the internasal air cells may be hyperpneumatized and extend into the cavernous portion of the internal carotid artery, raising the potential for vascular injury during endoscopy (90).

### 4.3 Preoperative considerations

Preoperative evaluation of sphenoid sinus hyperpneumatization requires undergo meticulous image analysis, as it can greatly influence not only surgical risk profiles, but also procedural approaches. High-resolution CT with isotropic voxel acquisition is pre- ferred for any imaging protocols, thus enabling accurate three-dimensional reconstruc- tions (91, 92). Preoperative CT data should be scrutinized in the coronal, axial, and sagit- tal planes to assess the full extent of the sinus cavity, the integrity of its walls, and the relationships of adjacent neurovascular structures. Multiplanar reconstructions using thin-slice protocols (0.3–0.5 mm slices) are crucial for the identification of subtle bony de- hiscences or atypical septal attachments in the case of suspected hyperpneumatization (93).

Three-dimensional (3D) modeling is gaining importance in the preoperative plan- ning especially for cases with complex hyperpneumatization patterns. Software-based segmentation algorithms can produce patient-specific 3D reconstructions that better il- lustrate spatial anatomy of sinus architecture (94). AR applications can be incorporated into navigation systems in the OR, allowing real-time overlying of these models on the surgical site during endoscopy (95). Studies have suggested a 25–30% decrease in neuro- vascular complications only when AR-assisted navigation is integrated in surgeries with hyperpneumatized sphenoid sinuses (96). Re-operative conversations within the multi- disciplinary team (neurosurgeons, otolaryngologists, and radiologists) should include an- atomical obstacles and surgical techniques. These conversations should account for hy- perpneumatization location and extent, neurovascular dehiscence presence, and surgical corridor exit angle & trajectory (97). For example, hyperpneumatized sinuses with lateral pneumatization into the pterygoid recess may require modified approaches to prevent vidian nerve injury. Likewise, cases with optic nerve canal dehiscence will need to be approached with special care so as to avoid accidental nerve injury during instrumenta- tion (55).

AI-powered tools in preoperative planning: understanding the future for sinus anat- omy. Convolutional neural networks (CNN) and support vector machine (SVM) can per- form radiomics-based analysis and extract quantitative features from CT datasets that may help predict hyperpneumatization related complications (98). Such algorithms ex- amine texture patterns, volumes of the sinuses, and bone density metrics, generating pre- dictive clues regarding anatomical variants that could complicate the surgical approach by computational analysis. AI-augmented preoperative planning shows a 20–30% im- provement in anatomical landmark identification compared to human assessment alone in pilots studies (99). This needs to be a collaborative, technologically driven synthesis of advanced imaging techniques and deep knowledge of sinus anatomy and potential for surgery. Advances in imaging modalities, as well as the wider use of integrating AI and 3D modeling technologies, will likely lead to improved diagnostic accuracy, reduced in- traoperative risk and, consequently, better outcomes in skull base surgery with respect to hyperpneumatized sphenoid sinuses. Realization of these im-aging, anatomical, and sur- gical planning benefits has been followed by an expanding body of primary literature that examines the detailed intricacies of sphenoid sinus hyperpneumatization (100). A wide range of studies have characterized these aspects, including anatomical differences, mo- lecular pathways, imaging technologies and clinical results (101). Key findings from re- cent studies regarding the anatomical, radiological, and surgical concerns associated with sphenoid sinus hyperpneumatization, as well as their clinical significance and potential avenues for future research, are summarized in **Table 1**.

**Table 1:** This table presents a curated summary of recent primary research studies investigat- ing sphenoid sinus hyperpneumatization.

Research Focus	Key Findings	Original Literature Insights	Clinical Implications	References
Anatomical Var- iability	Pneumatization varies in direction and extent, frequently extending into clival and pterygoid regions. Air-cell patterns correlate with craniofacial morphology and airflow dynamics.	CFD modeling suggests airflow- driven osteoclastic activation influ- ences air-cell extension.	Understanding anatomical variability aids in preopera- tive planning and reduces sur- gical uncertainty.	(50, 102, 103)
Neurovascular Risks	ICA dehiscence occurs in 15–25% of hy- perpneumatized sinuses; optic nerve canal dehiscence in 10–15%. Lateral pneumatization linked to higher cav- ernous sinus exposure.	Histopathological studies show in- creased RANKL/OPG expression in dehiscent ICA walls; nerve displace- ment confirmed by MRI tractog- raphy.	Preoperative identification of vascular dehiscence reduces the risk of hemorrhage and nerve injury.	(5, 104, 105)
Radiological As- sessment	High-resolution CT remains the stand- ard; DECT and PCCT improve bone- soft tissue contrast. 7T MRI reveals sub- tle mucosal remodeling and sinus wall irregularities.	AI-enhanced CT protocols demon- strate improved detection of thin- walled sinus regions, with 90% accu- racy in identifying dehiscent ICA ca- nals.	Refined imaging protocols en- hance surgical planning and reduce misdiagnosis of hy- perpneumatization as patho- logical erosion.	(40, 106, 107)
Molecular Mechanisms	Pneumatization driven by osteoclastic activity; RANKL/OPG dysregulation increases trabecular bone resorption. SHH/BMP signaling influences cranio- facial growth patterns.	Genomic analysis identifies RUNX2 polymorphisms associated with ex- cessive sinus pneumatization in de- velopmental syndromes.	Early genetic screening may identify patients at risk for hy- perpneumatization and re- lated complications.	(14, 15, 108)
Surgical Impli- cations	Hyperpneumatized sinuses complicate transsphenoidal approaches, distorting landmarks and increasing CSF leak risk. Lateral pneumatization affects vid- ian nerve trajectory.	Neuronavigation advancements (AR/VR overlays) reduce operative time by 15%; PATS protocol demon- strates improved orientation in hy- perpneumatized cavities.	Procedural adaptation and in- traoperative navigation re- duce complications in ana- tomically complex cases.	(109–112)
CSF Leak Dy- namics	Thinned skull base increases CSF leak risk by 3×; spontaneous leaks associated with posterior clival pneumatization.	Biomechanical studies reveal in- creased tensile stress at mucosa- bone interface in hyperpneumatized sinuses, correlating with leak inci- dence.	Preventive use of collagen- based grafts and hydroxyap- atite reinforcements mitigates leak risks.	(11, 113)
Symptomatic Presentations	Retro-orbital pain, facial numbness, and atypical headaches common; associated with nerve displacement and sinus aer- odynamics.	DTI studies demonstrate trigeminal nerve compression in pneumatized pterygoid recesses, correlating with facial pain severity.	Early identification and sinus decompression can alleviate nerve irritation and related symptoms.	(28, 114, 115)
Innovations in Sinus Surgery	AR/VR tools enhance sinus navigation; flexible endoscopic instruments im- prove visualization in irregular sinus cavities.	Clinical trials show 30% complica- tion reduction when AR-guided neuronavigation used in hyperpneu- matized sinus surgeries.	Adoption of AR-assisted tech- niques improves surgical pre- cision and reduces learning curve.	(116, 117)
Long-Term Im- plications	Unclear long-term outcomes; potential links to CSF pressure irregularities and craniofacial pain syndromes.	Pilot studies suggest pneumatiza- tion patterns may correlate with al- tered intracranial compliance and headache incidence.	Long-term follow-up with ad- vanced imaging may uncover predictive markers for chronic complications.	(22, 118–120)

It highlights critical findings across anatomical, radiolog- ical, molecular, and surgical domains, providing a detailed overview of the structural variations, clinical implications, and technological advancements associated with this anatomical variant. The insights summarized here offer a foundation for understanding the challenges posed by hyperp- neumatized sphenoid sinuses and underscore the importance of continued research in this evolving field.

## 5 Clinical significance in neurosurgical practice

The sphenoid sinus has a uniquely strategic location at the skull base serving as an important anatomical corridor for transsphenoidal surgical approaches to the sellar and parasellar compartments. Hyperpneumatization of this sinus adds considerable variabil- ity to sinus anatomy with clinical significance beyond anatomical interest. Sphenoid hy- perpneumatization, defined by expansion of the sphenoid sinus into surrounding struc- tures, including the clivus, pterygoid processes and anterior occipital bone, positively im- pacts surgical access, procedural safety and outcomes (83). The importance of sphenoid sinus hyperpneumatization to clinical practice results not only from its ability to change the position of anticipated anatomical landmarks, but also from the environments it gen- erates to provide both opportunities and risks of surgery. These anatomical variants re- quire more sophisticated surgical plans, refined preoperative imaging protocols, and dil- igence in understanding the pathophysiological underpinnings of sinus pneumatization (121).

The increasing use of EEAs has highlighted the importance of understanding sphe- noid sinus anatomy in detail, as hyperpneumatized patterns can be an asset but also pos- ing potential difficulties in surgical procedures involving this area. Excessive pneumati- zation can also facilitate an extended surgical corridor into the sellar region, potentially reducing the amount of bony removal necessary during surgical resection (122). Often, these benefits are compromised by the structural vulnerabilities engendered by thin or dehiscent bony walls, especially when such walls protect neurovascular structures (optic nerves, ICAs, and cavernous sinus). The unpredictable anatomical variations that accom- pany hyperpneumatization highlight the important role for multidetector high-resolution imaging, sophisticated neuronavigation systems, and meticulous surgical techniques ad- dressing the altered biomechanical matrix of the sinus (5, 123).

Research indicates that the clinical ramifications of hyperpneumatized sphenoid si- nuses go beyond surgical considerations alone. More recently, the association of exces- sive pneumatization with some craniofacial pain syndromes and potential changes of cer- ebrospinal fluid dynamics and biomechanics of the pituitary gland have been reported (25). Despite being early in their investigation, these hypotheses highlight the wider im- plications of sphenoid sinus anatomy for both clinical and research interpretation (124). Therefore, elucidating the role of hyperpneumatization in neurosurgical practices neces- sitates a multi-tiered inquiry including molecular pathways, biomechanical processes, clinical implications, and surgical innovation (50).

### 5.1 Impact on transsphenoidal approaches

The transsphenoidal approach, performed employing either microscopic or endo- scopic techniques, relies upon the consistency of certain anatomical markers within the sphenoid sinus. For normal anatomy, the midline sphenoid septum, sellar floor and OCR function as landmarks that orientate the surgeon during the operation (11, 125). But in hyperpneumatized sinuses, these landmarks can deviate from their normal location, re- gress, or cease to exist altogether. These changes disrupt the surgical workflow and intro- duce navigational uncertainty, particularly when approaching deep-seated sellar or par- asellar lesions (2, 36).

The intersinus septum, which normally provides a stable midline reference, is sig- nificantly variable in hyperpneumatized variants. In nearly 30% of hyperpneumatized sphenoid sinuses, the main septum is laterally displaced or eccentrically inserted into the bony walls of the carotid canal or optic nerve sheath (126). Such an atypical septal anat- omy can confuse surgeons who depend on conventional midline septal orientation and can increase the risk of iatrogenic injury. Furthermore, the hyperpneumatization process often produces accessory septa that do not have the normal structural integrity and are thin, fragile bony plates that easily fracture when handled. This fragility complicates transsphenoidal approaches, because fractured septal fragments can inadvertently trau- matize surrounding neurovascular structures (127). The OCR, an important anatomical landmark and demarcation of transition from the sphenoid sinus to the cavernous sinus, is noted to undergo major structural alterations in hyperpneumatized sinuses. In many instances, the recess looks elongated, laterally displaced, or distorted by irregular air-cell extensions (128). MRI tractography analyses have demonstrated that hyperpneumatiza- tion can shift the relative positions of the optic nerves and ICAs, sometimes resulting in the protrusion of these structures into the sinus cavity with little or no bony separation. Such protrusions lead to dangerous arenas where even slight instrument malpositioning can have disastrous consequences (129).

Hyperpneumatization can lead to thinning of the sellar floor and even dehiscence. Morphometric CT studies have shown that hyperpneumatization of the sellar floor is as- sociated with its average thickness being reduced more than 60% compared to that of nor- mally pneumatized sellar floors (130). This thinning not only raises the risk of CSF leaks but also deprives the pituitary gland of its mechanical support. Imaging follow-up post- operatively in these patients with significant sellar floor thinning has also documented descent of the pituitary gland into the sphenoid cavity, raising concern of related dysfunc- tion of the gland in these patients (131).

Recent molecular biological advances have started to enhance our understanding of the mechanisms behind sphenoid sinus hyperpneumatization. In hyperpneumatized var- iants, the regulated interaction between osteoblastic and osteoclastic activity, which con- trols paranasal sinus development, seems to be disturbed. Overexpression of RANKL alongside lower levels of osteoprotegerin (OPG) has been found in sinus mucosa from patients undergoing transsphenoidal surgeries (132). This increased osteoclastic action hastens the resorption of trabecular bone and leads to the hyperpneumatized cavities seen in hyperpneumatized sinuses (133). Studies have also implicated mutations in genes in- cluding RUNX2, which regulates osteoblast differentiation, and FGFR2, a major mediator of cranio-facial morphogenesis, in the pathogenesis of hyperpneumatization. Certainly, these findings implicate sphenoid sinus hyperpneumatization as potentially, in some cases, manifesting an individual genetic predisposition toward aberrant cranial base de- velopment (134). Sphenoid sinus hyperpneumatization becomes relevant for the transsphenoidal approach; thus, the biomechanical effect of sphenoid sinus hyperpneu- matization is significant. Finite element modeling (FEM) studies showed that thinning of the sphenoid sinus walls changes the distribution of mechanical loads forced through the cranial base (135). In hyperpneumatized variants, load-bearing areas are transferred to the posterior skull base, this leads to increasing mechanical stress within the clival region. This redistribution may explain the increased incidence of stress fractures and spontane- ous CSF leaks in patients with extensive pneumatization of the sinuses (136).

In addition, modified mechanical properties of the sinus walls may affect the stabil- ity of surgical reconstructions used to treat iatrogenic CSF leaks. The reduced adherence of graft materials when applied onto hyperpneumatized sellar floors has been corrobo- rated by experimental investigations, implying that in such situations, conventional au- tologous fat grafts and collagen patches may require adapted application techniques. The possible relationship between patterns of sinus pneumatization and pituitary gland bio- mechanics, by virtue of the direct anatomical relationship to the sellar floor, is yet another area of potential exploration (41). The anatomical variability associated with hyperpneu- matization warrants aquisition of modern image guided neuronavigation within tran- sphemoidal operations (110). These systems take preoperative CT and MRI datasets to generate 3D models of the sinus cavity and can provide real-time feedback on the posi- tion of an instrument during surgery. Neuronavigation is especially advantageous in cases of hyperpneumatized sinuses, where classic ananatomic landmarks may be unreli- able or missing (137).

Current neuronavigation systems utilize AR interfaces, projecting virtual reconstruc- tions of the sinuses onto the endoscopic display. Researchers examining AR-assisted neu- ronavigation for skull base surgery in a clinical study scribed a 27% decrease in intraoper- ative complications versus conventional guide methods. The decreased use of the soft- ware was attributed to better visualization of dehiscent bone areas and ectopically posi- tioned septa, in hyperpneumatized sinuses. Instead, intraoperative CT devices with neu- ronavigation systems provide for real-time reorientation due to anatomical displacements produced by tumor removal or CSF loss (95).

Hyperpneumatized sphenoid sinuses can be detrimental and have led to new surgi- cal techniques to maximize procedural outcomes. One of these innovations is as follows, by utilizing pneumatization-adapted transsphenoidal surgery (PATS) which is a protocol that adapted the path of surgical trajectories based on preoperative imaging features. PATS protocols integrate sinus morphometry via the navigation algorithm to account for anatomical distortions during planning. Initial findings indicate that PATS leads to re- duced operative durations as a result of fewer intraoperative recalibrations and improved anatomical predictability (9, 138). A novel method includes the usage of hydroxyapatite-reinforced bone grafts for the reconstruction of thinned sinus walls after tumor removal. These grafts offer greater me- chanical stability than conventional fat or fascia grafts and have shown long-term struc- tural stability in hyperpneumatized sinuses. Nor has the concept of using biodegradable collagen matrices for mucosal regeneration been ignored, and, most recently, surgeons have attempted to employ this material in order to avoid postoperative collapse of the enhancing mucosal flap, especially in extremes of pneumatization (139). Hyperpneumati- zation of the sphenoid sinus has previously been considered a purely anomalous anatom- ical variant, however new studies have suggested that extensive pneumatisation may be associated with some craniofacial pain syndrome. Synonyms since pain is often sug- gested in cases of persistent facial pain and headache, trigeminal nerve compression, caused by expansion of the sinus cavity into the recess of the jawbone. Changes in posi- tion of the pituitary gland that occur with thinning of the sellar floor have also raised the possibility of endocrine sequelae, but evidence remains limited (140). The evolution of new neurosurgical techniques and imaging technologies will certainly improve our knowledge of sphenoid sinus hyperpneumatization and its clinical significance. Future research should prioritize the development of predictive models that estimate hyperpneu- matization severity from craniofacial morphometry data, as well as the genetic basis that causes such anatomical variation. Furthermore, machine learning algorithms applied to radiomic data could offer new biomarkers for the preoperative identification of high-risk variants (141). The future of neurosurgical management of hyperpneumatized sphenoid sinuses will truly be paved with a combination of these anatomical, molecular, biome- chanical and clinical insights. With greater understanding, surgical algorithms will likely utilize ever increasing constructs of patient anatomical data to augment procedure safety and efficacy (142).

### 5.2 Risks and complications

Hyperpneumatization of the sphenoid sinus, while generally considered a benign anatomical variant, presents neurosurgical concerns that arise from the challenge to skull base integrity. Any aberrant pneumatization of the clivus, pterygoid processes, or greater wings of the sphenoid has the potential to alter the biomechanical stability of the cranial base, cause thinning or dehiscence of protective bony walls, and predispose the surgical approach to increased risk of CSF leaks or neurovascular injury. If this were the extent of the potential adverse effects, the implications would be timely corrective surgical measures. However, these structural shifts or anomalies can correlate with various symptomatic presentations like headache and facial pain. The extensive nature of these complications clearly warrants a thorough examination of their potential pathophysiological mechanisms, clinical significance, and existing approaches to diagnosis and management (143).

Neurovascular injury is perhaps the most significant complication associated with hyperpneumatization, specifically in relation to injury to the ICA during surgery. ICA canal dehiscence is present in 15–25% of cases of the hyperpneumatized sphenoid sinus, with complete bony exposure in 3–7% of cases (6, 30). The anatomical reason for this vulnerability relates to excessive osteoclastic activity through which the expression of RANKL is upregulated and OPG is suppressed in the sinus mucosa (144, 145). RUNX2 polymorphisms could negatively impact bone integrity of the sphenoid area (146). The association of intra-operative ICA injuries experienced when the anterior septal attachments extend to the carotid canal reinforce the importance of high-resolution CTA preoperatively to characterize at-risk configurations (147, 148). Optic nerve canal dehiscence, which can occur in 10–15% of cases, also increases the risk of iatrogenic optic nerve injury. Bony attenuation at areas like the opticocarotid recess can reduce wall thickness to below 0.2 mm and place the nerve at risk for sustained mechanical injury or thermal injury. DTI research has shown that altered fiber pathways will be found in these scenarios that correlate with a chronic compressive stress (149). Involvements into lateral or pterygoid recesses may add pressure to cranial nerves such as III, IV, V1, V2, VI, or the vidian nerve in an occupying manner that presents with chronic orbits facial pain, and/or chronic orality with or without autonomics. Trigeminal neuralgia has even been described with pneumatization to the foramen rotundum (16).

The only route for prevention is detailed preoperative imaging. Dual energy CT and high-angle CTA will require thin slice (0.3 mm) CT so that critical neurovascular components can be adequately mapped (150). In the case of hyperpneumatization, dual energy CT (DECT) will be helpful to help delineate thinned hyperpneumatized bone, and pathologic erosion. The integration of AR integrated neuronavigation is capable of providing improved intraoperative orientation even with the distortion of anatomical landmarks (151). In situations where dehiscence is noted, it may be possible to employ low-speed drilling instruments (e.g., diamond burrs) in situations of thermal injury and the Doppler ultrasonography can preserve intraoperative vascular mapping (152). An MRA may be helpful postoperatively to report on vascular integrity or the presence of pseudoaneurysm (153).

CSF leaks are a more serious consideration. These leaks will typically occur at the area of the skull base to the location of the skull base where the cortex, at the sellar floor and/or clival recess area, thins out (154). Further, risk for CSF leaks is at least doubled (2-3 fold) with hyperpneumatized anatomy versus what considered normal sinus anatomy (155). Finite element models demonstrate significant stress concentrating due to an intracranial pulley from the thinning of the cortex to below 0.5 mm thickness (58, 156). These risks are only going to be magnified by any form of inflammatory process. CFD-documented alterations in air flow patterns increase shear stress in the sphenoethmoidal recess stimulate mucosal remodeling and have been shown to upregulate matrix metalloproteinases (MMPs) and decrease collagen deposition weakening the dura (157, 158). The symptoms of CSF rhinorrhea can present very subtly and mimic allergic rhinitis. Associated symptoms can include orthostatic headache, a metallic taste, or visual disturbances (159).

High-resolution CT cisternography and intrathecal gadolinium-enhanced MRI imaging provide sensitive diagnostic modalities to localize the leaks (160). The standard for repair is autologous fascia lata or collagen grafts. For larger defects, vascularized nasoseptal flaps will provide a solid closure and integration into the mucosa (161). Hydrogel sealing systems are now marketed and experimental stem cell filled scaffolding are being utilized but not yet formally studied for dural regeneration (162). The symptomatic syndromes will also include chronic headaches, facial pain, and visual disturbances. Headaches are usually deep, retro-orbital, or occipital which can closely resemble primary headache syndromes. Such headaches could be the result of trigeminal nerve impingement, or mucosal inflammation or compression of the optic nerve. DTI studies are showing microstructural changes in the affected nerve in imaging of symptomatic patients (163–165). Facial pain is common if the pterygoid recess is involved, especially if the vidian nerve is displaced. In some cases, decompressing this area helped alleviate symptoms (166, 167). Visual symptoms including diplopia or optic neuritis have been reported if the optic nerve where compressed in patients with canal dehiscence, likely as a result of ischemia or direct mechanical insult to the nerve (168).

In conclusion, sphenoid sinus hyperpneumatization may no longer be hypocritical as simply an incidental anatomical variation. It deserves a much deeper understanding of the pathogenesis, radiological findings, and clinical impacts as it relates to skull base neurosurgery. Further research into the unique genetic and molecular regulators of pneumatization signals such as FGF and SHH signalling pathway could be invaluable to developing knowledge on disease processes. Future directions that utilize AI morphometric and radiomic assessments for earlier risk stratification, and biomechanical assessments of 3D printed sinuses, could help direct surgical developments. Greater understanding of these processes in turn could improve safety and care for those undergoing neurosurgical intervention (1, 169).

## 6 Management strategies

The management of hyperpneumatization of the sphenoid sinus presents a particu- larneurosurgical challenge due to the anatomical unpredictability and surgical vulnera- bilities evoked by excessive pneumatizations. The large air cells, which at times extend to the clivus, pterygoid processes, or greater wings of sphenoid, conjoin to form a surgical terrain unique in distorted spatial relations and obliterated or missing bony partitions ad- jacent to neurovascular elements of paramount significance. This anatomical deviations demand an individualized evidence-based strategy of management with respect to the preoperative, intraoperative and postoperative phases (170). As evident from this paper, optimal results will be reliant on complex imaging protocols, straightforward surgical modifications, and close postoperative vigilance, all with directive assistance from a re- search and technology-intensive multiprofessional milieu (171).

### 6.1 Preoperative planning

Consequently, preoperative planning of hyperpneumatized sphenoid sinuses in CLG must follow a multi-pronged strategy, ensuring anatomical fidelity yet preserving with facets of procedural insight. In hyperpneumatized cases, dynamic alteration of sinus morphology should guide individual assessment, as well as the consideration of detailed imaging analysis, anatomical risk stratification, and a multidisciplinary approach for planning (172).

Radiological investigation of the hyperpneumatized sphenoid sinuses which pre- sents a complexity in evaluation because; unlike ordinary protocols, there are subtle struc- tural changes it being a rare entity, thus demanding detailed investigation of the same. High-resolution multidetector computed tomography (MDCT) is the anatomical gold standard, with modern CT scanners capable of achieving isotropic voxel sizes of 0.2–0.3 mm to create three-dimensional reconstructions with exquisite spatial resolution (71). However, hyperpneumatized variants are intricate and supplementary methods are needed. New spectral pho-ton-counting CT (PCCT) utilizes energy-de-pendent attenua- tion coefficients within the area of interest to map *in vivo* trabecular micro-architecture at submillimeter resolution, ultimately providing enhanced sensitivity in differentiating a hyperpneumatized bone from pathological osteolysis (173). Risk stratification is being transformed with the use of algorithms based on bone texture analysis derived from pre- operative imaging. Such analyses depend on algorithms for which machine learning models are trained using very large-scale sinus CT datasets to evaluate bone homogeneity, cortical porosity, and fractal dimensions to describe sites at high risk for intraoperative fractures or dural dehiscence (174). Early clinical studies demonstrated that applying these machine learning techniques to bone texture analysis within the pre-operative work- flow improved the prediction of surgically significant anatomical variants by 20–25% and such information offers a rationale for actionable insights which could help inform surgi- cal planning (175, 176).

MRI is key to understanding adjacent neurovascular anatomy adj neurovascular structures and the integrity of the surrounding soft tissues. Black-blood MRI techniques, which attenuate signals from vascular lumens and accentuate walls of vessels, proved particularly useful in the identification of CA dehiscensce and early vessel wall pathology (177). There has also been recent application of diffusion-weighted magnetic resonance spectroscopy (DW-MRS) further characterizing sinus mucosal changes by quantifying re- spective molecular signatures abroad of chronic mucosal stress and dysregulated dynam- ics of airway pneumatization (178).

In summary, hyperpneumatization presents with some anatomical complexity that should be discussed in detail in a multidisciplinary team preoperatively. Planning and collaborative discussion of the imaging findings would facilitate anticipation of what challenges should be anticipated procedurally, and contingency strategies amongst the neurosurgeons, otolaryngologists, neuroradiologist, and anesthesiologists (179). Discus- sions should ideally address the presence of anatomical variances (i.e. extensions of the clival recess, aberrant septal attachments, pneumatization of the pterygoid process) that- may complicate surgical navigation. In order to make this collaborative process easier, innovative computational tools such as personalized anatomical atlases have been devel- oped These are produced from patient-specific imaging datasets and allow interactive ma- nipulation in a 3D sinus model, simulating appeals and predicting anatomical shifts due to tumor resection or CSF leak repair for a surgical team (180).

Patients should be able to specify in preoperative counseling visit whether they have hyperpneumatized sinus anatomy and to what extent and should understand (in evi- dence based terms) the risk this anatomy poses. In general practice, such discussions should arise not only in the context of the risk of standard surgery but also with respect to longer operative times, elevated risks of CSF leak and subsequent risk for transient postoperative neurologic symptoms like altered facial sensation or headache. The benefit of using patient-specific 3D models is that during these sessions, the patients realized bet- ter understanding of the surgical procedure and develop less perioperative anxiety (41).

**Figure 2:**
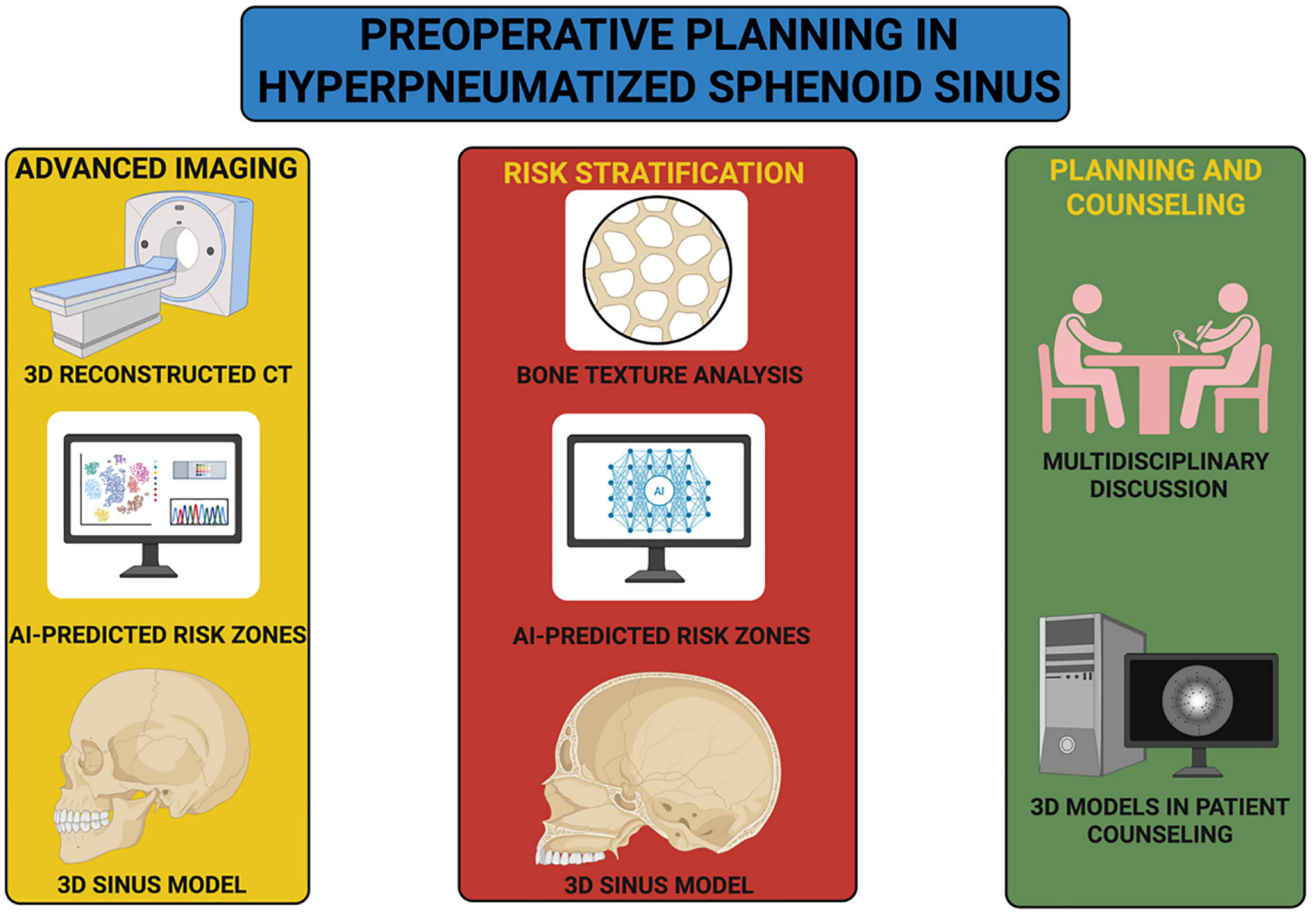
Structured preoperative planning workflow for hyperpneumatized sphenoid sinus.

In support of this patient-centered and technology-integrated approach, **Figure 2** intends to outline a structured preoperative planning workflow that synthesizes imaging precision, risk modeling, and multidisciplinary coordination to enhance both surgical preparedness and patient understanding.

The diagram illustrates an integrated clinical pathway for managing anatomically complex sphenoid sinus pneumatization. The process begins with advanced imaging techniques, including high-resolution CT and AI-augmented analysis to generate personalized 3D sinus reconstructions and identify high-risk anatomical zones. These data inform risk stratification through bone texture analysis and machine learning–based prediction of structural vulnerabilities, such as dural dehiscence or ICA exposure. The workflow culminates in multidisciplinary discussion involving neurosurgeons, otolaryngologists, neuroradiologists, and anesthesiologists, accompanied by patient counseling aided by interactive 3D models. This structured approach aims to enhance surgical planning, support shared decision-making, and reduce perioperative uncertainty.

### 6.2 Surgical techniques

Hyperpneumatized sphenoid sinuses in Publications should be addressed surgically with methods, specifically adapted to the particular constraints of such anatomic malfor- mations, malleable bony covers andremodelled neurovascular interactions and the need for greater interdisciplinary communication and collaboration. The erratic course of such pneumatized air cells call for surgical flexibility, vigilance inspite of surgical progresses, and the application of innovative surgical technologies (181). Hyperpneumatization cre- ates and imperfect space which the Atlas and inverse forms of the aforementioned tech- niques must be adapted to. The primary modification is to recalibrate the anatomy re- peatably in the operative navigation systems. In cases of hyperpneumatized sinuses, standard landmarks such as the sphenoid septum and opticocarotid recess may be located in their normal locations, but visualization can be difficult (182). In these situations, ma- chine-learning–augmented neuronavigation platforms have greatly improved an-atomi- cal localization. These systems combine real-time imaging data analysis on a continuous basis and identify and visualize the anatomical transformations that occur during bonet thinning associated with pneumatization (183). When selecting access corridors in hy- perpneumatized sinuses, consideration should be given to the distribution of air cells and the location of critical neurovascular structures. A new approach ‘lateralized sphenoid corridor technique’ serves as a way to remove lesions present in highly pneumatized si- nuses with extreme lateralized pneumatization. Intraoperative Doppler ultrasound has since been used to identify the vidian canal and sphenopalatine artery as landmarks in approaching pneumatized pterygoid recesses, however this technique was carried out prior to these ultrasound-derived landmarks being described (10).

Given the fragile nature of hyperpneumatized sphenoid walls, we can expect to per- form bone removal with fine instruments and fine-controlled forces. An approach that can prevent accidental penetration through thinned osseous partitions is sensorized re- sistance-based adaptive microdrills. These drills tab auto-rotate torque modulate when they encounter the resistance of low-density bone and reduce the risk of a dural perfora- tion. Tools specifically designed for cranial sinuses, such as angle endoscopes with stere- otactic overlay systems have also been employed within complex pneumatizedconfigura- tions. To facilitate safer dissections around important landmarks (i.e., cavernous sinus and optic canal) (184).

In hyperpneumatized sphenoid surgeries, preserving mucosal integrity is crucial in order to reduce postoperative complications. Surgical techniques which aim to preserve architecture such as mucosal-sparing dissection contemporaneous with blunt dissection and hydro dissection agents have demonstrated smooth sinus mucosal architecture in the most pneumatized of cavities. To a comparative reduction of postoperative sinus dysfunc- tion and more rapid reepithelialization of the mucosa *in-situ* (185).

### 6.3 Postoperative care and follow-up

The management of patients with hyperpneumatized sphenoid sinuses in the post- operative period must be adapted to the vulnerabilities that excessive pneumatization provides. The major goals in this period are the early identification of complications, preservation of sinus function, and anatomical and functional assessment (105).

Hyperpneumatized skull bases are structurally deficient whereby patients are sus- ceptible to postoperative CSF leaks that can be associated with significant morbidity. Sur- veillance protocols must contain serial nasal endoscopic examinations to evaluate surgical disruption sites and early indicators of CSF leak as chronic rhinorrhea or enhanced nasal output. Of these, transnasal intrathecal fluorescein testing has demonstrated the highest sensitivity for occult leaks, with sensitivities to detect a leak of >95%, when high-resolu- tion MRI cisternography is utilized in conjunction (186). Dynamic nasal airflow monitor- ing is a new noninvasive assessment of postoperative sinus function. This method uses micropressure sensors to measure air-pressure differentials in the sphenoethmoidal recess and provides indirect evidence of CSF leaks by detecting abnormal airflow patterns with- inunsealed dural defects (187). Mucociliary clearance dynamics (altered) with increased mucosal (surface) area for infection—hyperpneumatized sphenoid sinuses are predis- posed to development of postoperativeinfections. Preventive strategies should focus on optimal sinus aeration and regulating mucosal health. Nasal irrigations that disrupt bio- films that include xylitol-based solutions are shown to reduce postoperative sinus infec- tions by destroying bacterial biofilms and preventing bacterialcolonization of exposed mucosal surfaces (188). Compared to conventional non-contrast enhanced sequences, con- trast-enhanced T1-weighted MRI sequences should be integrated into the postoperative imaging armamentarium to evaluate for infectious complication, possibly involving the out-of-sinus cavity. Infections of the pneumatised clival or pterygoid recess can be intra- cranially spreading and should be treated expeditiously (antibiotic spectrum guided by intra-operative cultures when available) (189).

Due to the close proximity of hyperpneumatized sphenoid sinuses to important neu- rovascular structures, we recommend that postoperative neurological assessment extend beyond standard visual and cranial nerve exams. In follow-up assessments, multimodal evoked potentials (MEPs) are able to detect subclinical deficits in cranial nerve function, most notably in-patients with optic nerve canal pneumatization (190). Studies showed that the detection of early-stage optic neuropathy was sensitive and non-invasive in individu- als with predominated sinus pneumatization using pattern-reversal visual evoked poten- tials (PR-VEPs) (191). Hyperpneumatization may have implications for the stability of the skullbase in long-term follow-up. High-tech complementing imaging with4D flow MRI revealed deranged cerebrospinal fluid dynamics in patients of hyperpneumatized si- nuses that could pave the way for idiopathic intracranial hypertension or spontaneous CSF leaks’years down line (192). Reassuring intradural ICP episodes and periodical im- aging should be reserved to those with prominent posterior or clival pneumatization (193).

Over time, imaging, surgical instrumentation, and personalized medicine will grad- ually improve and revolutionize the non-invasive management strategies of sphenoid si- nus hyper-pneumatization. It would be helpful to further explore the different molecular mechanisms governing this process of sinus pneumatization, and in particular whether hypoxia-inducible factors (HIFs) affect the activity of osteoclasts at the sinus walls (194, 195). Furthermore, there could potentially be individualized risk-based surgical al- gorithms introduced in preoperative planning software combined with AI-based ana- tomic mapping algorithms allowing materialized predictive tracts (196).

With further studies, the development of clinical, anatomical, and biomechanical workup will be essential in order to optimize surgical techniques, improve patient out- comes and define sphenoid sinus hyperpneumatization as an anatomical variant of im- portance (124).

## 7 Future directions and conclusions

The sphenoid sinus was once considered an inert anatomical space of limited clinical significance. Pneumatization patterns, particularly when excessive, afford them surgical topographies counter to traditional guidelines, necessitating a problem-solving mentality, adaptability, and multimodal strategy. However, the literature review indicated that our understanding of sphenoid sinus hyperpneumatization remains fraught with a number of questions. This review illustrates the incapability of current approaches with the modernimaging modalities, molecular biology, computational modeling, and advanced surgical techniques that are leading us to a future in which these questions may be an- swered (40). It does so by subsequently providing areas of continued exploration to focus on, emerging technologies likely to strongly influence their clinical application, and mus- ings on the enduring scientific interest in this remarkable organ.

### 7.1 Research gaps and unanswered questions

To date, anatomical counterparts of sphenoid sinus hyperpneumatization have been documented with increasing frequency but remain unclassified using a uniform diagnos- tic taxonomy. However, similar terminology denominative conchal, presellar, sellar, and postsellar patterns—current in writing but does not study the morphometric characteris- tics that differentiate distinctly one of each other. This non-standardized characterization limits the ability to associate variants with clinical results, making more challenging deci- sions during the surgery and also comparative data from institution to institution. Such performance based grading system should be developed universally based on volumetric, morphometric, and biomechanical parameters. A modern variation of sucha system would likely be capable of not only assigning an LOC stage to the patient based on the multislice CT images, but also of stratifying severity with complex radiomic analyses, and metrics like ratios of sinus volume to bone-thickness and the extent of pneumatization into contiguous skull base structures (197).

In courage, wherever classification fails, the long run consequences of sphenoid si- nus hyperfect-one-ness are still yet to be completely gripped. While short-term perioper- ative complications such as CSF leaks and neurovascular injury have been extensively characterized, long-term sequelae have remained relatively understudied (198). Does hy- perpneu-matization affects the compliancy of cranial base leading to spontaneous intra- cranial hypotension as risk factor? Does chronic mechanical stress on the neurovascu- larunits explain late-onset cranial nerve complications? Further prospectivelongitudinal studies with imaging and neurocognitive assessments at established intervals may eluci- date these possible associations, and inform preventive interventions (199).

Otherwise, molecular basis of hyperpneumatization Also remain to be the underex- plored frontier. Introduction: Pneumatization is a multifactorial process regulated by sig- naling from the epithelium, osteoclastic activity and cranial base growth patterns. Pre- clinical data indicate that dysregulation of RANKL/OPG signaling pathways may play a role to be responsible for exaggerated boneresorption in regions of hyperpneumatization (200). The regulatory networks downstream of this process, and above all how theyare modulated by hypoxia-inducible factors (HIFs) and fibroblast growth factor 10 (FGF10), remain poorly characterized. Investigation of these pathways utilizing high-resolution single cell RNAsequencing and CRISPR-based gene editing models may unveil molecu- lar signatures that serve as predictive markers for the development of a pathological sinus (201).

An avenue that establishes the correlation of hyperpneumatization and sinonasal physiologythis needs exploration. Several studies mentioned earlier based on CFD indicate that excess pneumatization alters airflow dynamics within the sphenoethmoidal re- cess area which might play a role in inducing the mucosal inflammation, barosinusitis or headache syndromes (202). But those simulations are still theoretical, with no clinical data to support them. Such theory was distributed at modeling efforts, pro-spective studies with *in vivo* (real-time)nasal-airflow monitoring may help find if such aerodynamic dis- turbances are clinically relevant (203).

### 7.2 Technological advancements and future innovations

The field of skull base surgery is evolving into a dynamic portal in the sky, where technological developments are rapidly changing the diagnostic and treatment para- digms. Novel technologies like imaging, navigation and intraoperative monitoring can significantly enhance the handling of hyperpneumatized sphenoid sinuses (204).

#### 7.2.1 Augmented reality and virtual reality in surgical planning

AR and VR platforms capable of integration in the preoperative planning have moved from proof-of-concept to practical application. AR systems including marker- based tracking, or surface registration algorithms are already delivering some value for intraoperative orientation and augmenting anatomical landmarks in the operative field (205). We are at the tipping point between future AR systems and holographic sinus re- constructions that are projected directly into the endoscopic viewport. The holograms were generated from high-resolution CT datasets and could potentially be updated dy- namically to match the position of a instrument in real time, if this were done in reality, thus providing a more intuitive, spatially accurate presentation of the3D architecture of the sinus cavity (206).

While virtual reality is mainly used for surgical education at this time, it may soon prove to be essential within the patient-centered area of pre-operative simulations. VR platforms that use physics based anatomical modeling could enable surgeons to practice navigating hyperpneumatized cavities in silico, practicing potential complications such as intraoperative hemorrhage or septal dislocation. Pilot studies have demonstrated that VR-based rehearsal sessions are able to decrease operative time byas much as 18%, high- lighting the more tangible applications of this type of technology in this domain (207).

#### 7.2.2 AI-driven imaging and predictive analytics

AI applications in sinus imaging represent one of the most promising frontiers in Its brightest future for skull base surgery may lie in applications for imaging ofthe sinus. Radiomic analysis, as a novel technique based on quantification of patterns of texture, shapes, and intensity on imaging datasets, has become a new tool to assess and predict potential hyperpneumatization degree and associated surgeons’ risk. These may in turn be followed by deep learning CNNs trained on large-scale imaging repositories to detect anatomical signatures of high risk variants — such as carotid artery dehiscence or sellar floor thinning (208).

But not on anatomical characterization, the concept of predictive analytics also ex- tends. An area of recent research has focused on integrative (prognostic) models to predict long-term outcomes by integrating imaging features with traditional patient de- mographics, genetic markers, and clinical history into a unified model. Such models, if confirmed in heterogeneous patient cohorts, may provide individually tailored risk fac- tors to surgeons, and guide decision making for choice of proc-l strategy (209, 210).

Endoscopic instrumentation is evolving with new imaging and navigation technol- ogy. Through novel adaptive optical systems, next-generation endoscopes are now able to dynamically adjust both focus length and image resolution in real continuum to best visualize patency in anatomically non-conforming cavities. Creating scalpel-formatted multi-lumen endoscopes containing intraluminal, embedded probes for nerve stimulation also offers an additional layer of safety when resecting adjacent hyperpneumatized lat- eral walls containing cranial nerves (211).

### 7.3 Expanding clinical perspectives

The implications of sphenoid sinus hyperpneumatization are not restricted to skull base surgery. The functional consequences of changed sinus anatomy may be widespread, spanning specialties from neurology, to oto-laryngology, to pain medicine (212).

For instance, the suspected linkage of hyperpneumatization to headache syndromes has not yet been definitively established. The persistence of retro-orbital discomfort in some patients with pneumatized clival recesses is contained in retrospective studies that corroborate potential trigeminal nerve irritation. Perhaps studies combining neurologists and headache specialists might clarify these associations and further the role of sinus anatomy in the physiopathology of craniofacial pain (213). Moreover, hyperpneumatiza- tion may also contribute to alterations in biomechanics, which may affect alterations in cerebrospinal fluid dynamics that may play a role in diseases such as idiopathic intracra- nialhypertension and spontaneous intracranial hypotension. Studies using 4D flow MRI to clarify CSF flow patterns through different sinus morphologies may offer some clues towards cranial base related subsystems that contribute to overall regulation of ICP (214). One area of growing importance in sinus management is regenerative medicine. Tissue-engineering approaches, including a bioengineered mucosal graft with patient-de- rived stem cells, could conceivably be employed to fortify thinned sphenoid walls or re- store injured mucosa in the future (215). Pilot studies have as yet validated the feasibility of this approach for frontal sinus repair with case for future expansion to defects related tosphenoid hyperpneumatization (9).

### 7.4 Conclusions

The sphenoid sinus is one of the most enigmatic paranasal sinuses and a microcosm of anatomical surgical and scientific interest. What initially appeared to be a passive ana- tomic variant, hyperpneumatization, has subsequently emerged as a dynamic, clinically relevant event that challenges many of the tenets of established wisdom about cranial base morphology and skull base surgery. We have navigated these hallowed lumens of sphe- noidsinus, however meticulous, understanding the anatomical details, radiological ex- pression, surgical ramifications of sphenoid sinus hyperpneumatization like never be- fore. These observations highlight how important it is to take a cooperative, innovative approach to such anatomical variation, which reduces morbidity. Now, with the advance- ment in imaging modes, well-honed surgical techniques and the possibilities, computa- tional modes are helping us immensely in tuning hyperpneumatization of sinus and in treating them better. But even with those advances, we still don’t know enough. Mecha- nisms to date explaining pneumatization, biomechanical effects of sinus cavity expansion, and long-term effects of reduced or absent cranial nerve function for the remainder of an individual’s life have differed, and one correlate of this lack of adaptive ease may be the multitude of molecular pathways augmented by biomephysical and biomechanical fac- tors that remain richly fertile domains of future research.

In many ways, the anatomy of the sphenoid sinus is a microcosm for the larger pur- suit of anatomical knowledge. It serves as a reminder that small ana-tom-ical curiosities can have mighty clinical consequences, overturning well established assumptions and leading to new revelations. The sphenoid sinus remains at the end of this paper what it has always been: the hidden chamber at the heart of the skull base, breathing soft whis- pers of secrets to those who will listen. And while there have been broader mysteries and more to come, the multitude of layers of billions of singular economies sending their tem- porary gifts out is too much to overcome, but as the spectres of future higher minds await the riddle of the dead and how immediate, as if we knew exactly what human tissues lay impounded inside the irony of the anatomical histories.
