# Correction: Sphenoid sinus hyperpneumatization: anatomical variants, molecular blueprints, and AI-augmented roadmaps for skull base surgery

**DOI:** 10.3389/fendo.2026.1781516

**Published:** 2026-01-16

**Authors:** Andra Ioana Baloiu, Florin Filipoiu, Corneliu Toader, Razvan-Adrian Covache-Busuioc, Octavian Munteanu, Matei Serban

**Affiliations:** 1 Doctoral School, “Carol Davila” University of Medicine and Pharmacy, Bucharest, Romania; 2 Department of Anatomy, “Carol Davila” University of Medicine and Pharmacy, Bucharest, Romania; 3 Department of Neurosurgery, “Carol Davila” University of Medicine and Pharmacy, Bucharest, Romania; 4 Department of Vascular Neurosurgery, National Institute of Neurology and Neurovascular Diseases, Bucharest, Romania; 5 Department of Medical Research, Puls Med Association, Bucharest, Romania

**Keywords:** hyperpneumatization, skull base anatomy, transsphenoidal surgery, cerebrospinal fluid leakage, neurovascular injury, advanced imaging, artificial intelligence

Affiliation “Doctoral School, “Carol Davila” University of Medicine and Pharmacy, Bucharest, Romania” was omitted from the paper. This affiliation has now been added for the author “Andra Ioana Baloiu”.

The original version of this article has been updated.

